# Advancing 3D Engineered In Vitro Models for Heart Failure Research: Key Features and Considerations

**DOI:** 10.3390/bioengineering11121220

**Published:** 2024-12-03

**Authors:** Elisa C. H. van Doorn, Jorik H. Amesz, Olivier C. Manintveld, Natasja M. S. de Groot, Jeroen Essers, Su Ryon Shin, Yannick J. H. J. Taverne

**Affiliations:** 1 Translational Cardiothoracic Surgery Research Lab, Department of Cardiothoracic Surgery, Erasmus Medical Center, 3015 GD Rotterdam, The Netherlands; e.vandoorn@erasmusmc.nl (E.C.H.v.D.); j.h.amesz@erasmusmc.nl (J.H.A.); 2 Department of Cardiology, Cardiovascular Institute, Erasmus Medical Center, 3015 GD Rotterdam, The Netherlands; o.manintveld@erasmusmc.nl (O.C.M.); n.m.s.degroot@erasmusmc.nl (N.M.S.d.G.); 3 Department of Molecular Genetics, Erasmus Medical Centre, 3015 GD Rotterdam, The Netherlands; j.essers@erasmusmc.nl; 4 Division of Engineering in Medicine, Department of Medicine, Brigham and Women’s Hospital, Harvard Medical School, Cambridge, MA 02139, USA; sshin4@bwh.harvard.edu

**Keywords:** translational research, heart failure, *in vitro* modeling, cardiac tissue engineering, 3D models, myocardial remodeling, cardiac fibrosis, extracellular matrix, biomimetic scaffolds, cell maturation

## Abstract

Heart failure is characterized by intricate myocardial remodeling that impairs the heart’s pumping and/or relaxation capacity, ultimately reducing cardiac output. It represents a major public health burden, given its high prevalence and associated morbidity and mortality rates, which continue to challenge healthcare systems worldwide. Despite advancements in medical science, there are no treatments that address the disease at its core. The development of three-dimensional engineered *in vitro* models that closely mimic the (patho)physiology and drug responses of the myocardium has the potential to revolutionize our insights and uncover new therapeutic avenues. Key aspects of these models include the precise replication of the extracellular matrix structure, cell composition, micro-architecture, mechanical and electrical properties, and relevant physiological and pathological stimuli, such as fluid flow, mechanical load, electrical signal propagation, and biochemical cues. Additionally, to fully capture heart failure and its diversity *in vivo*, it is crucial to consider factors such as age, gender, interactions with other organ systems and external influences—thereby recapitulating unique patient and disease phenotypes. This review details these model features and their significance in heart failure research, with the aim of enhancing future platforms that will deepen our understanding of the disease and facilitate the development of novel, effective therapies.

## 1. Introduction

Heart failure (HF) is a complex clinical syndrome characterized by structural and functional abnormalities of the myocardium, leading to inadequate cardiac output [1]. HF currently represents a major public health burden, affecting 1–3% of the global population [2] and having an average annual mortality rate of 33% [3]. Despite extensive efforts to improve HF therapy, prognosis remains poor, with treatment generally focusing on symptom management rather than disease modification [4]. There are no disease-modifying therapies available other than left-ventricular assist devices or heart transplantation for end-stage cases. Moreover, nine out of ten drug candidates fail during clinical trials [5], contributing to the decline in the emergence of novel and effective therapies [6]. The lack of preclinical HF models with high resemblance to human *in vivo* conditions is partly to blame for this phenomenon [7]. Traditionally, drug development and testing have relied on animal models and *in vitro* cultures, both of which have greatly contributed to scientific insights on cardiac disease [8]. Yet, animal models inherently suffer from inaccuracies in interspecies extrapolation and are increasingly subjected to ethical objections, whilst *in vitro* platforms often fail to grasp the full complexity of human biology [8]. It therefore remains essential to employ more accurate *in vitro* constructs to advance our understanding of HF pathophysiology and expedite the discovery of new therapeutic interventions.

*In vitro* models serve as cellular imitations of the human body, designed to emulate specific organ segments rather than replicate the intricacies of an entire organism. These models currently span a wide range of complexity, from simple single-cell studies to advanced organoids and micro-physiological systems that recapitulate the properties of miniaturized organs [9,10,11,12,13]. Rationally, ideal *in vitro* models should closely resemble human *in vivo* cardiac features, replicating the disease phenotype and its underlying causality to enable clinical translation. However, the degree of *in vivo* resemblance of an *in vitro* model is typically proportional to the complexity of its design, with the inclusion of a greater number of factors required for higher fidelity [13]. The pursuit of new therapeutic options for HF often necessitates the use of both simple and complex *in vitro* models in tandem, each offering unique insights into cardiac pathophysiology and the mechanisms of potential drug candidates. The specific advantage of three-dimensional (3D) engineered constructs, compared to simpler cellular platforms and two-dimensional (2D) models, resides in their capacity to manage model complexity more explicitly and precisely tune the specific properties that define their features.

Attaining high-level *in vivo* resemblance in 3D-engineered *in vitro* models for HF remains a challenge, and several key factors must be considered. These involve accurately mimicking extracellular matrix (ECM) features including matrix (1) composition, (2) architecture, (3) mechanical compliance, and (4) electrical conductivity. In addition, consideration must be given to the proper selection of cellular factors, such as (5) cell source and cell type, (6) cell type ratio, and (7) cell maturity. Other model features to be considered include (8) dynamic factors continuously imposed on cardiac tissue and vessels *in vivo*, such as cyclic strain and fluid shear stress, (9) interactions with other organ systems, (10) environmental and external influences, and (11) gender- and age-related disparities. Given these complexities, this review aims to provide a comprehensive outline of the key characteristics needed to develop 3D-engineered *in vitro* models that accurately mimic HF. A detailed description of 3D-engineered model platforms (Table 1) is beyond the scope of this review, as it has been elaborately discussed elsewhere [14,15,16,17,18].

## 2. Cardiac Remodeling in Heart Failure

HF involves various pathological tissue remodeling processes, characterized by reshaping, renewing, and reorganizing of tissue architecture in response to adverse triggers, such as unfavorable genetic predispositions, infections, mechanical overload, ischemia, oxidative stress, and iatrogenic injuries [19,20]. Initially adaptive, this remodeling becomes harmful over time, leading to systolic impairment or diastolic failure [20]. Tissue remodeling in HF has both structural and functional dimensions. Structural remodeling, such as cardiac fibrosis (CF), hypertrophy, and dilatation, involves changes in the size, shape, and ECM composition of cardiac tissues, altering the heart’s architecture. Functional remodeling impacts cardiac performance and is often due to calcium mishandling, ion channel alterations, and neurohumoral activation [21]. Effective HF modeling should encompass these biomechanical processes to allow for a comprehensive study of remodeling. 

CF involves the excessive accumulation of fibrous connective tissue in the heart, disrupting its structure and function [22,23]. It presents as interstitial, replacement, or perivascular fibrosis, contributing to impaired systolic and diastolic functions in HF with both preserved and reduced ejection fractions by stiffening the myocardium and reducing its contractile power, respectively [24,25]. Numerous efforts have been made to accurately model CF *in vitro* and identify strategies to modify fibrotic remodeling, e.g., through the use of cardiac spheroids [26] or mechanically tunable hydrogels [27]. Increased work and stress result in hypertrophy, which can be classified into two forms: concentric and eccentric [28,29]. Concentric hypertrophy is often observed in HFpEF, where increased wall thickness and stiffness impede proper diastolic filling. Eccentric hypertrophy is more typical in HFrEF, where chamber expansion compromises systolic function [30,31]. Dilative remodeling commonly occurs in chronic conditions involving continuous volume overload, such as valve regurgitation, as well as in ischemic injury following myocardial infarction, or genetic predispositions associated with dilated cardiomyopathies [32]. *In vitro* cardiac hypertrophy is typically modeled by applying afterload to engineered tissue, as illustrated by Stenzig et al. [33] and Hirt et al. [34], whereas cardiac dilatation may be mimicked by applying repetitive stretch [35].

Functional remodeling in HF includes calcium mishandling, where disruptions in calcium ion regulation impair muscle contraction and relaxation [36,37]. Dysfunction in key proteins including the ryanodine receptor, SR Ca^2^⁺-ATPase (SERCA), and phospholamban may lead to impaired calcium release and reuptake from the sarcoplasmic reticulum (SR) within cardiomyocytes (CMs), contributing to both systolic and diastolic dysfunction, as well as pathological remodeling [38,39]. *In vitro*, CM cultures are a common tool for assessing calcium mishandling and proteins involved, as illustrated by Minor et al. [40] through the use of genetically manipulated induced pluripotent stem cells (iPSCs). In addition, alterations in sodium, potassium, and calcium ion channels drive electrical remodeling, heightening the risk of arrhythmias and impaired cardiac function [41,42]. Neurohumoral activation, through overactivation of the renin–angiotensin–aldosterone (RAAS) system and sympathetic nervous system, may further exacerbate HF by promoting vasoconstriction, fluid retention, and structural remodeling [43].

For further elaboration on the complexities of HF remodeling, both structural and functional, readers are referred to detailed reviews that explore the underlying pathophysiology and potential interventions [22,23,29,31,32,39].

**Figure 1:**
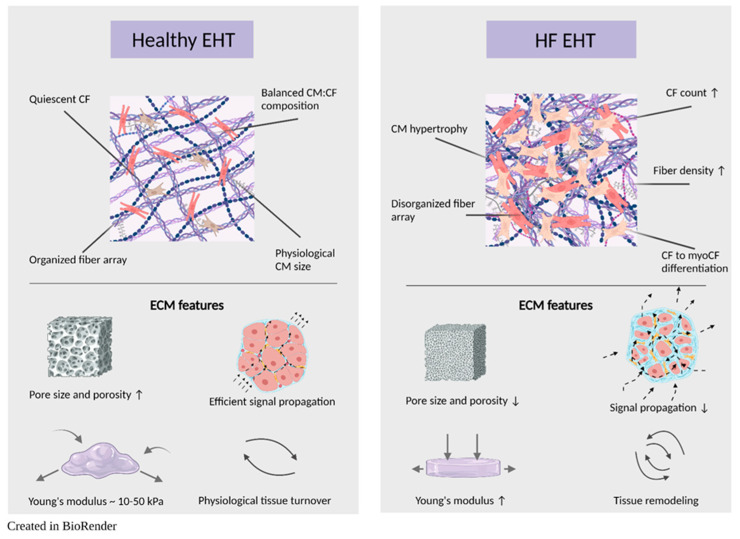
Comparison of ECM features in healthy and HF engineered heart tissues (EHTs).

Given the complexity of HF and the multifaceted nature of cardiac remodeling, developing sophisticated models is essential for gaining deeper understanding of pathophysiology and exploring novel therapeutic avenues. Advanced HF modeling is predicated on a dual-faceted strategy. First, the *in vitro* construct should attain optimal *in vivo* mimicry at its core. Depending on the research objective, emulating inter-organ interplay, such as the cardio-renal axis or the cardio-brain connection, may be crucial to attaining comprehensive physiological responses. Second, factors that induce HF remodeling should be incorporated to faithfully replicate the structural and functional alterations observed *in vivo.* Consequently, the initial section of this review focuses on accurately modeling the myocardium as it exists *in vivo*, while the subsequent sections delve into model features, key factors, and considerations for HF modeling. Figure 1 highlights the key differences between the extracellular matrix structures in healthy and HF-affected myocardium.

## 3. Extracellular Matrix Features

### 3.1. Composition

*In vivo*, CMs and other cardiac cells attach to a collagen-based ECM, which provides physical support. The ECM is a highly dynamic structure, constantly undergoing remodeling to control tissue homeostasis. ECM components interact with cells to regulate a multitude of functions, including cell attachment, proliferation, migration, and differentiation [44]. Minor changes in ECM content and organization may greatly influence cellular structure and function, and subsequently the (cardiac) tissue as a whole [45]. The success of an engineered *in vitro* model hinges on its ability to mimic intricate ECM characteristics of native tissues [46,47,48]. This involves mirroring biological ECM features in terms of biocompatibility, morphology and degradability.

### 3.2. Biocompatibility

The human cardiac ECM mainly consists of structural proteins, predominantly collagen fibers, elastin fibers, and fibronectin, as well as basement membrane proteins like laminin, and non-structural components including glycosaminoglycans and proteoglycans [49]. Collagen type I is the most abundant ECM protein in the heart, strengthening the matrix [45]. Collagen type III and elastin provide entropic elasticity, allowing the heart muscle to reversibly expand and relax with every cardiac cycle [50]. Proteoglycans together with glycosaminoglycans form the main constituents of the ECM’s ground substance, an amorphous gel-like structure that may be considered a natural glue to fill up the extracellular space and attach cells to ECM-components [51]. Fibronectin is a fibrillar connective protein that conveys information between cells and fibers [52]. Laminins compose the basement membranes of cells, providing them with mechanical support as well as biological signals, either by interacting with cell surface proteins or indirectly by trapping growth factors [51]. Through these interactions, they trigger and control cellular functions, thus playing roles in communication and ECM functionality. Furthermore, many ECM-associated proteins are present that, while not part of the complex network mesh, are nonetheless pivotal to cardiac ECM function. These include growth factors, cytokines, signaling molecules, hormones, and enzymes involved in fiber crosslinking. 

**Figure 2:**
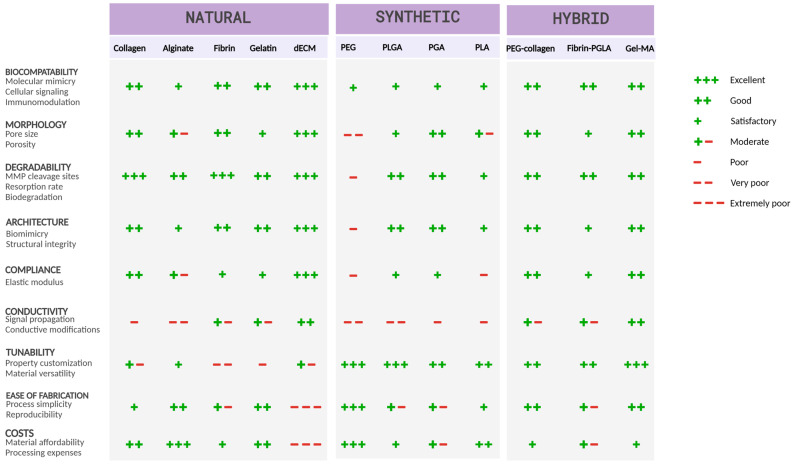
Comparison of scaffold properties in cardiac tissue engineering. Decm = decellularized extracellular matrix, PEG = polyethylene glycol, PGLA = poly(lactic-co-glycolic acid), PGA = polyglycolic acid, PLA = polylactic acid, Gel-MA = gelatin methacryloyl. Created in BioRender.

Currently, no technology can fully replicate the intricate structure of *in vivo* cardiac ECM. Simulating a healthy ECM before introducing cues that trigger HF remodeling, or for use in control models, ensures that any observed differences are solely due to the induced HF triggers and not to an initially inappropriate matrix that lacks biocompatibility. At present, hydrogels are the most widely used biocompatible ECM matrices or scaffolds for engineered cardiac tissue [53]. These hydrogels can be classified into three categories: natural hydrogels (e.g., collagen, fibrin, gelatin, alginate), including decellularized extracellular matrices (dECM); synthetic hydrogels (e.g., polyethylene glycol (PEG) or poly(lactic-co-glycolic acid) (PLGA)); and hybrid hydrogels, which are a blend of both [54,55] (Figure 2). Natural hydrogels closely mimic native ECM, while synthetic hydrogels provide greater versatility for customization. Hybrid hydrogels aim to integrate the advantages of both [53]. dECM scaffolds may be deemed superior for achieving perfect molecular ECM mimicry. However, frequently used natural polymers, PEG- or PLC-based matrices, and hydrogels also provide a native-like environment for cardiac culture [55]. An ideal scaffold should at least provide a sufficient number of cell-binding sites to permit physiological tissue density, and a certain degree of biomimetic diversity to support various cell functions.

### 3.3. Morphology

Cardiac ECM is characterized by a high porosity (>90%) and pore diameters of around 20 μm [56]. This is necessary to facilitate cell migration and nutrient penetration throughout the structure [57]. Since capillary diffusion is difficult to reproduce *in vitro* without the engineering of vasculature, macroporous scaffolds with pores of at least 100 μm and porosity > 90% are required for *in vitro* CM cultures to establish physiological delivery of oxygen and nutrients [58,59]. For *in vitro* HF modeling, the use of macroporous matrices becomes vital as the disease is marked by significant ECM changes due to remodeling [60]. These changes also affect porosity and eventually alter the matrix structure as a whole. HF-associated remodeling, including CF and hypertrophy, typically entails the deposition of ECM proteins and CM enlargement, respectively, which collectively lead to reduced intercellular space and overall tissue porosity. Consequently, the inherent macroporosity of suitable scaffolds, such as hydrogels, is crucial for accurately modeling pathological changes in HF, rendering them popular candidates for cardiac *in vitro* engineering.

### 3.4. Degradability

*In vivo*, cardiac ECM undergoes continuous remodeling through cleavage by matrix metalloproteinases (MMPs), which helps preserve and regulate ECM composition and structure while also releasing biologically active molecules, such as growth factors. MMP activity is physiologically low but may be heightened during repair or remodeling processes as occurs in HF [45]. Designing tissue scaffolds should take MMP cleavage into account, enabling a certain degree of scaffold turnover and adaptation to prevailing (patho)physiological circumstances. These may incite CF, hypertrophy, dilation or other structural remodeling events that accompany HF. Matrix scaffolds based on natural polymers inherently possess a higher degree of biodegradability, while synthetic matrix materials often exhibit slower rates of decomposition [61]. Among synthetic scaffold materials, PLGA and PGA are renowned for their highly biodegradable nature and tunable characteristics [62].

### 3.5. Architecture

Cardiac cells and ECM components are intricately organized into a specific 3D architecture within the living human heart, defining both physiological properties and pathological traits in disease states [63]. While 2D monolayers provide the most simplified representations of cardiac tissue *in vitro* [64], most 3D engineered modalities including hydrogel models, organoids and heart-on-a-chip platforms attempt to more faithfully recreate the myocardial layer as it occurs *in vivo* [65,66,67,68]. This native architecture encompasses features such as the parallel alignment of CMs, a specific spatial distribution of ECM fibers (e.g., the interwoven pattern of collagen), and a rich network of blood vessels, lymphatic vessels, and interstitial cells [69]. In HF research, the significance of mimicking cardiac in *vivo* architecture is highly dependent on the research objective at hand. Engineered 3D models based on simpler architectural designs, such as basic cell-laden hydrogels, allow for more controlled manipulation of parameters improving reproducibility while being less resource-intensive. Pertaining to HF investigation, such constructs facilitate the study of cellular behavior during remodeling and the impact of specific interventions with relative ease and reproducibility [47]. They may serve as a starting point for initial screenings and proof-of-concept studies. Models exhibiting architectures of medium complexity including organoids [70,71] and more advanced engineered tissues such as those generated from bioprinting techniques [72,73], vascularized 3D engineered tissues [53,74], and tissues used within heart-on-a-chip constructs [65,75,76] can better disclose multicellular crosstalk, cell–matrix interactions, vasculature interplay, and tissue development. Moreover, these models offer enhanced control over architectural matrix design, including the alignment of CMs through patterning structures [48]. Finally, they also replicate physiological responses that are more reminiscent of the native cardiac environment, including contractility kinetics, electrical signal propagation and fluid flow dynamics within a vasculature network. This underscores the importance of making a well-balanced decision in model design, as higher-complexity models demand increased resources, time, and specialized expertise to develop and sustain while often posing challenges to reproducibility. Of note, while blood vessel mimicry has been a primary focus in cardiac engineering, the role of lymphatic vessels in cardiac health and disease, including wound healing and fibrosis prevention, underscores their importance. Incorporating lymphatic vessel emulation into cardiac models could greatly enhance architectural fidelity [77]; however, it remains a challenging and rather unfamiliar field. At the far end of the spectrum, dECM scaffolds provide the most *in vivo*-like architectural framework, being derived from a natural source and offering an accurate appraisal of cardiac structure. These platforms are fit for more sophisticated research queries on HF remodeling and its impact on cardiac ECM architecture specifically [49]. dECMs can, for example, be used to explore the regenerative potential of stem cell or other cell-based therapies by repopulating the matrix and evaluating subsequent tissue function restoration, facilitation of neovascularization, and attenuation of pathological remodeling [49]. Finally, the utilization of dECMs offers a promising avenue to investigate the interplay between immune cells and cardiac remodeling, shedding light on the emerging understanding of the pivotal role played by inflammatory responses in HF pathophysiology [78]. *In vivo*-like cardiac architecture can also be synthetically recreated using 3D printing techniques. For instance, Mohammadi et al. [79] employed soft lithography to fabricate scaffold sheets featuring microgrooves that facilitate cardiac cell attachment and guide cell directionality. These scaffolds were wrapped around a central mandrel, resulting in a conically shaped model that mimics the shape of left ventricle. 

Fabrication methods play a crucial role in determining the organization of cells within cardiac engineered tissue [80]. Three primary techniques commonly employed include molding, seeding, and 3D printing. Molding refers to the suspension of cells in a prepolymer solution, which forms the matrix scaffold material. This solution is then crosslinked within a mold, creating a cellularized construct with cell distribution influenced by the properties of the prepolymer. Seeding involves populating preformed matrices—either natural (dECM) or synthetic—with cells using techniques such as electrospraying, electrowriting, or electrospinning. Finally, 3D printing allows for cells to be incorporated into a bioink, which is used to fabricate a 3D construct [80].

### 3.6. Mechanical Compliance

Mechanically, the compliance of an *in vitro* engineered scaffold for cardiac cell culture should approach that of native cardiac tissue (Young’s modulus ~10–50 kPa) [81,82]. Matrix compliance serves as an insoluble cue for many cells, influencing cell morphology, gene and protein expression profiles, cellular organization, as well as differentiation [81]. CMs cultured on relatively stiff substrates show decreased beating activity and lack mature, well-striated sarcomere structures [81]. In addition, various studies have reported that increased mechanical ECM stiffness is associated with unprompted transition of quiescent cardiac fibroblasts (CFs) into myofibroblasts (myoCFs) [83,84]. Contrarily, matrices that are too flexible are unable to provide sufficient mechanical resistance for the power stroke of the myosin filaments in the shortening-contraction cycle, resulting in inefficient contractions [81,85]. Achieving accurate myocardial tissue compliance is therefore crucial for correctly modeling HF, as changes in tissue stiffness not only affect healthy tissue similarity, but also reflect important tissue remodeling. Diastolic HF is typically marked by fibrotic remodeling and concentric hypertrophy which both increase tissue stiffness and lessen tensile strength [86]. This in turn impairs ventricle wall relaxation and causes elevated filling pressures, leading to failure [87]. Utilizing a scaffold that is initially too rigid would fail to capture this remodeling effect, essentially simulating a pathological state from the outset. In the case of systolic HF, myocardial compliance may either increase or decrease, contingent on the underlying etiology. Ischemic events, which incite myocardial infarction (MI) and associated ischemic cardiomyopathy, are marked by reparative fibrosis. This fibrosis stiffens the heart muscle due to ECM fiber deposition and contracture of myoCFs [10,88]. However, dilated cardiomyopathies—which also diminish pumping force—are characterized by a decline in compliance caused by thinning of the muscular wall and expansion of individual CMs [89]. The selection of matrix compliance ought to be guided by the intention to capture specific remodeling effects or to employ increased or decreased compliance as a tool for simulating pathological conditions.

### 3.7. Electrical Conductivity

Human myocardium operates as a functional syncytium, using electrically coupled myocytes to coordinate cardiac contraction by passing action potentials between individual cells. *In vivo*, electrical coupling mainly involves gap junctions between CMs, dependent on cell maturity. In tissue-engineered constructs, however, matrix conductivity significantly influences electrical conduction due to often suboptimal cell–cell coupling *in vitro*, which can be compensated with enhanced scaffold conductivity [90]. Recent studies confirm that employing electrically conductive scaffolds enhances native-like electrical properties. Conductive nanomaterials, such as carbon nanotubes (CNTs), gold nanoparticles (including gold nanowires (GNWs) and gold nanorods (GNRs)), and graphene oxide (GO), are gaining popularity [91,92,93]. Scaffolds embedded with GNRs facilitate signal propagation and improve electrical coupling by promoting connexin-43 expression, the primary gap junction protein, and synchronized beating of CMs [94]. In addition, electroconductive polymers (ECPs) like polypyrrole (PPy), polyaniline (PANI), and poly(3,4-ethylene dioxythiophene) (PEDOT) offer tunable conductivity and are frequently also used in scaffolds [91,92,93]. HF is marked by cardiac electrophysiological disturbances, including a decrease in conduction velocity which could significantly contribute to the development of atrial and ventricular arrhythmias [95]. These changes are attributed to HF-related remodeling that disrupts gap junctions and negatively impact ion channel physiology. It is crucial to model the electrical properties of the matrix scaffold and the engineered tissue accurately to account for the effects of remodeling on electropathology. Failure to do so would undermine the ability to assess CM excitation and contraction kinetics, limiting the potential of these models for investigating the true pathogenesis of HF and developing new treatments affecting these electrophysiological parameters.

## 4. Cellular Features

In designing engineered tissue, it is crucial to carefully select appropriate cell types, their origins, ratios, density, and maturity to accurately replicate the *in vivo* cellular architecture.

### 4.1. Cell Source and Cell Type

The heart is a complex organ composed of numerous cell types that collectively form the entire cardiac apparatus, including CMs, CFs, vascular endothelial cells (VACs), pericytes, smooth muscle cells (SMCs), migrating immune cells, autonomic nerve cells, and stem cells also known as cardiac progenitor cells (CPCs) [16]. Cardiac (patho)physiology is orchestrated by the inherent electromechanical coupling of these cells and their continuous crosstalk, and single cell type dysfunction leads to different pathologies than multicellular failure. When modeling *in vitro*, one can choose from several cell sources to establish cardiac cultures, including primary cells, embryonic stem cells (ESCs) and iPSCs. Primary cells are directly obtained from cardiac tissue, usually animal-based, in contrast to stem cells which need to undergo differentiation. Engineered tissue cultures should ideally be based on human cell sources in order to attain genetic mimicry.

Utilizing single-cell or models with fewer cell types can highlight isolated roles of different cell types in remodeling. In the past, CM monocultures were preferred, with CFs systematically eliminated due to their tendency to overproliferate and cause undesirable tissue changes. However, recent research has shown that the co-culture of non-myocytes, especially CFs, is crucial for the maturation of cardiac tissue. As such, interactions among multiple cell types promote the development of native-like cellular structures and functionality [63,96,97,98]. Cells have proven to survive longer, adhere firmer and express higher levels of maturity in tissue constructs that consist of multiple cell types [16,99]. Also, mixed cell type compositions may be deemed vital when used as platforms for drug testing, considering that therapeutic compounds sort effects through all cardiac cell types rather than CMs alone [63]. However, *in vitro* culturing more than one cell type is challenging, with increasing complexity being directly related to cell type number. Different cell types respond differently to specific *in vitro* tissue properties, including matrix stiffness, scaffold composition, oxygen supply, and cell-cell and cell-ECM contacts. This demands meticulously tuned tissue properties and environments to maintain high-quality engineering multicellular cultures [15,99]. To effectively model HF, consideration of cell type composition within engineered cardiac tissues is crucial. HF-induced remodeling is often orchestrated by multiple cell types working in concert. For instance, CF is primarily initiated by CFs, but negatively affecting CM function [24]. Additionally, immune cells play significant roles in HF remodeling through immune reactions such as macrophage-mediated promotion of inflammation and fibrosis, and T cell cytokine release. In genetic cardiomyopathies such as dilated cardiomyopathy (DCM), hypertrophic cardiomyopathy (HCM), restrictive cardiomyopathy (RCM), and arrhythmogenic cardiomyopathy (ACM), primary genetic defects within CMs result in structural and functional abnormalities, whereas CFs instigate fibrosis and immune cells drive associated immune responses [100]. The most widely used cell type combinations to study HF are CM-CF cocultures or combinations of CMs with mesenchymal stem cells, as these cell types significantly contribute to remodeling following pathological triggers [97]. However, depending on the type of HF being modeled, additional cell types may be necessary. For instance, ECs are beneficial for modeling ischemic events, while adipocytes are valuable for modeling diabetic cardiomyopathy.

### 4.2. Cell Type Ratio and Density

Adequate cell type ratios and densities should be considered when assembling multicellular tissue constructs. Although CMs occupy 70–80% of the volume of the human heart, they only constitute 20–30% of cardiac cells by number [101,102]. CFs usually reside in a quiescent state *in vivo* but may be activated into collagen-producing myoCFs post-injury to prompt fibrotic tissue repair. Inconveniently, CFs cultured *in vitro* tend to over-proliferate and quickly differentiate into myoCFs, thereby skewing the CF:CM ratio [94]. This may result in unintentional fibrotic tissue simulation. In order to maintain functional and native cardiac-like cell type ratios, CF-CM constructs should preferably be cultured in 3:7 ratios [97] (or 30% non-CMs), as a foundation for physiological and/or pathological modeling. A relative increase in CF count inherently leads to heightened tissue stiffness and the promotion of conduction blocks, offering a potential platform for simulating HF-associated fibrotic remodeling.

Moreover, cell densities should permit accurate simulation of spatial cell features and contribute to cell-mediated compaction of the ECM. The latter refers to the process by which cells exert mechanical forces on the surrounding ECM, leading to its compression and densification. This phenomenon stands as a hallmark of functional cardiac tissue as it contributes to structural integrity, electrical coupling, cell–ECM interactions and contractile function [97]. It is essential however to strike a balance, avoiding overcrowding to maintain appropriate direct cell–cell and cell–ECM interactions, thus fostering native-like micromilieus [47,65]. The appropriate cell density hinges on the chosen scaffold type. For instance, in hydrogel constructs, a range of 1–50 × 10^6^ cells/mL can establish effective cell-cell connections without overcrowding, depending on the cell source and mixture composition [47,48,65,97]. However, with PLGA-based polymers or fibrin-based natural scaffolds, the optimal cell density may range between 1–5 × 10^5^ cells/mL [103] and up to 30 × 10^6^ cells/mL [104], respectively. Therefore, meticulous optimization of cell type density is crucial for each new experimental configuration as no universal ideal cell density has been determined for each scaffold type, owing to inter-center variations. Typically, in hypertrophy, the size of CMs increases. However, to approximate the hypertrophic effect on tissue, one could increase CM density, thereby simulating the augmented mass of the heart muscle whilst elevating CF density could mimic fibrotic alterations.

### 4.3. Cell Maturity

Cell maturity is crucial for accurately modeling HF, particularly when using iPSCs [105]. Maturation involves profound phenotypic and functional changes that are required for a cell to attain its fully operative state. Adult CMs are cylindrical, multinucleated cells with large nuclei, characterized by highly organized sarcomeres, well-developed sarcoplasmic reticula (SR) and transverse tubules (T-tubules). They are well-aligned and are extensively interconnected by intercalated disks composed of tight junctions, desmosomes, and gap junctions [106]. In contrast, immature CMs tend to be smaller, mononucleated, and roundly shaped, lacking proper cell alignment [63]. Also, immature CMs possess fewer mechanical and electrical junctions, as well as a reduced number of mitochondria surrounding the nucleus. Furthermore, they have shorter sarcomeres, poorly developed SRs, and hardly any T-tubules which causes slow calcium handling. Functionally, immature cells lack full differentiation, limiting their ability to express key maturation-related genes essential for proper function in health and disease. As a result, they fail to accurately mimic HF pathophysiology and cannot replicate adult cell responses to adverse stimuli like hypertrophy, necrosis, apoptosis, or gene expression changes observed in mature cardiomyocytes during HF progression. In addition, observed drug effects may not accurately reflect *in vivo* responses, further reducing their utility in drug development efforts. Continuous efforts are directed towards enhancing the maturity of iPSC-CMs through mechanical, electrical, and biochemical stimulation, along with cellular factors and 3D engineering, in order to closely emulate the native environment [107,108]. Advancing beyond the sole assessment of cell maturity, the maturation of engineered cardiac tissue should also be considered. In this context, certain hallmarks of adult myocardium serve as invaluable benchmarks. Adult myocardium exhibits distinctive features, including a positive response on inotropic agents [7], a positive force–frequency relationship (FFR) and post-rest potentiation (PRP) [97]. The former denotes heightened contractile force as heart rate escalates in healthy conditions, while the latter signifies enhanced contractility following a period of quiescence—a testament to CM’s capacity to efficiently manage calcium cycling within the sarcoplasmic reticulum (SR). Moreover, ventricular myocardium *in vivo* displays a refractory period (RP) typically ranging between 200 and 300 milliseconds, regulating the rhythm and integrity of cardiac contractions. Lastly, *in vitro* tissues exhibit better maturity as evidenced by declining excitation thresholds (ET) and increasing maximum capture rates (MCR), metrics pivotal in gauging tissue maturation [97]. These parameters collectively serve as discerning indicators of tissue maturity, crucial in the comprehensive evaluation of engineered cardiac constructs. 

## 5. Model Features

### 5.1. Dynamic Modulation in Cardiac Tissue Engineering and Disease Modeling

The environment of cardiac tissue in the human heart is highly dynamic, continuously influenced by numerous factors that affect organ structure and function. This includes cyclic mechanical strain (stretch and compression/strain forces) imposed on the heart during its contraction cycle, fluid shear stress (from laminar blood flow), electrical gradients from action potential propagation and biochemical stimuli. Several studies have shown that the mimicry of mechanical forces and electrical pacing into engineered cardiac microtissues are essential to significantly improve cell maturity, functional cell–cell coupling and tissue architecture [80,97,109,110]. For instance, a study by LaBarge et al. [111] demonstrated that a custom mechanical stimulation bioreactor system that replicates cardiogenic stretch patterns significantly enhances the sarcomeric organization of CMs. Another study conducted by Zhao et al. [97] found that weekly 1 Hz step-up pacing of engineered cardiac tissue led to a positive FFR, enhanced PRP, lower ET, and higher MCR compared to other pacing protocols or no pacing at all.

Mechanical stimulation in engineered cardiac tissue is usually achieved through cyclic stretch, compression, or both [112]. Electrical pacing is typically accomplished by positioning an inert electrode, often made of graphite or platinum, on both sides of the cardiac tissue construct within the medium, enabling the passage of an electrical current pulse through the tissue, a concept referred to as field stimulation. Several key factors must be taken into account when employing electrical stimulation, including pulse width, pulse frequency, and stimulation intensity. In human-based constructs, pulse width and stimulation intensity are contingent upon the size and composition of the engineered tissue. Typically, the pulse frequency is set at 1 Hz, equivalent to 60 beats per minute [80]. In contrast to field stimulation, point stimulation can better mimic electrophysiological responses by generating electrical wavefronts that glide through the tissue, rather than an all-at-once stimulation burst. However, point stimulation is more challenging to implement and is less commonly used in current cardiac engineered *in vitro* models.

Furthermore, incorporating pulsatile hemodynamic forces on engineered cardiac tissue, thereby simulating pulsatile blood flow, has recently been found to enhance CM maturation *in vitro* especially compared to static cultures [113]. For example, in a study by Kolanowski et al. [113], hiPSC-CMs cultured in a microfluidic system under controlled pulsatile flow conditions showed significantly enhanced sarcomere organization and increased expression of cardiac-specific markers. 

Lastly, biochemical composition of cell culture media may also greatly influence tissue structure and function. For cardiac cell and tissue culture, the selection between glucose-based medium and FFA (Free Fatty Acid) medium hinges on the primary objectives of the study. The main energy source for CMs *in vivo* is fatty acids, which account for 60–70% of their energy production. Glucose-based medium, however, provides rapid energy through glycolysis and is commonly used for standard cardiac cell cultures to promote initial growth [114]. FFA medium such as DMEM, on the other hand, mimics the *in vivo* environment more closely, supporting better maturation and functional properties of cardiac tissues. For physiological relevance and functional studies, FFA medium is therefore preferred for cardiac tissue engineering [115,116]. In addition, hormones and growth factors such as insulin-like growth factor 1 (IGF-1), transforming growth factor-beta (TGF-β), thyroid hormone, and triiodothyronine (T3) have shown to be important for cardiac tissue development and maturation. Other factors such as epidermal growth factor (EGF), fibroblast growth factor (FGF), and endothelin-1 (ET-1) have been identified to influence cardiac cell proliferation and differentiation [117]. For example, Katili et al. [118] demonstrated that co-culturing hiPSC-CMs with CFs and ECs, along with the application of IGF-1 and TGF-β, significantly enhanced the maturation process of engineered cardiac tissues. Furthermore, a study by Wong et al. [119] showed that combining T3 and IGF-1 in the culture medium improved the maturation and functionality of engineered cardiac tissues, demonstrating the synergistic effects of these biochemical cues. 

Tunable dynamical factors within cardiac tissue platforms can also be leveraged to model HF states, such as diminished blood flow during cardiac ischemia and subsequent blood flow recovery during reperfusion, by adjusting media flow and oxygenation [10,120]. Cyclic mechanical strain can be tuned to mimic volume and/or pressure overload by inducing high preload and afterload, respectively, activating intracellular pathways that prompt adverse remodeling and HF [35,65,68]. Electrical stimulation can be employed to simulate pathology through high-frequency pacing [121] or irregular pacing [122], leading to tachyarrhythmia-induced cardiomyopathy [123] or electrical remodeling. In addition, hormones elevated in HF, such as norepinephrine, angiotensin II, and endothelin-1, can also impact cardiac function and remodeling. Incorporation of these factors into culture media can therefore aid high-level mimicry of pathological changes contributing to HF [25]. Lastly, research has shown that in heart failure (HF), cardiomyocytes (CMs) undergo a metabolic shift to primarily utilize glucose due to altered energy demands and reduced oxygen availability [114]. In research by Abdurrachim et al. [124], the use of a high-glucose medium was employed to simulate the metabolic conditions of HF *in vitro*, demonstrating how increased glucose utilization can impact cardiac cell function and model HF states effectively.

Ideally, experimental models should therefore consist of platforms in which mechanical, physical, electrochemical, and biochemical factors can be integrated and regulated in order to better recapitulate the dynamic microenvironment of cardiac tissue *in vivo* and accurately mimic disease. The advent of heart-on-a-chip platforms has revolutionized the integration of dynamic stimuli into cardiac research, marking a swiftly evolving domain that has seen notable progress in recent years [14,92], including applications in HF modeling. For instance, McCain et al. [68] as well as Kong et al. [65] imposed cyclic stretch and compression overload, respectively, onto engineered cardiac tissue on-a-chip to induce pathological remodeling, leading to diastolic dysfunction. This method was used to investigate structural changes, electrical remodeling, and calcium handling in the failing myocardium. In addition, Rexius-Hall et al. [125] investigated the effects of a myocardial infarction border zone, which they created on a chip using a microfluidic-induced oxygen gradient. Their study focused on the electromechanical functions of the engineered cardiac tissue and its gene expression profiles.

### 5.2. Interaction with Other Organ Systems

Cardiac function *in vivo* is regulated by interactions with other organ systems involving neurohormonal, renopulmonary, and inflammatory signaling. Examples include systolic and diastolic blood pressure fluctuations, sympathetic and parasympathetic stimulation from the autonomic nervous system, pulmonary vascular resistance, hormonal factors released by RAAS affecting heart rate and contractility force, and inflammatory cytokines [126]. When recapitulating the heart *in vitro*, emphasis is usually not laid on mimicking the interactions with other organ systems. However, accurately modeling human cardiac (patho)physiology ideally includes exposure to inter-organ interactions. Inflammation, in particular, plays a pivotal role in the pathogenesis of HF, as immune cells infiltrate the cardiac tissue in response to systemic and local inflammatory signals [127]. Isolated cardiac models may overlook this complexity unless specifically integrated into the model, albeit usually in simplified forms [99]. In addition, the evaluation of therapeutic compounds, including HF drugs, is complicated by the fact that these are primarily metabolized by the liver and kidneys. Therefore, *in vitro* cardiac models lacking functional liver and kidney tissues are inherently limited in assessing pharmacokinetics [128]. Multi-organ models (e.g., body-on-a-chip platforms) offer a promising approach to replicate the intricate *in vivo* milieu, allowing for a more accurate representation of the complex interplays between different organs and their physiological processes [75,129,130,131]. These platforms are typically developed by connecting microfluidic chip systems, such as heart–liver or heart–kidney models, which are frequently used in pharmacokinetic studies. However, each organ and tissue type requires a unique microenvironment, coordinated signaling pathways, metabolic exchanges, specific culture media and flow rates, as well as dynamic cues to achieve full maturation and *in vivo*-like characteristics. Consequently, integration on microfluidic platforms remains a complex challenge [14]. Notably, *in vitro* models can also benefit from studying isolated cardiac (patho)physiological processes and drug effects without the interference of other organs [12]. In doing so, simple models can offer valuable insights depending on the research aim at hand.

### 5.3. External Stimuli

The human body is consistently exposed to environmental features, and increasing evidence supports the role of factors such as day/night cycles, seasonal changes, sunlight exposure, geographical location, altitude, and air quality as important determinants of cardiac health and disease risk. Lifestyle choices, including diet, exercise, and smoking also significantly impact the risk, progression, and severity of HF [132]. Most cardiac platforms do not account for these factors, but they should be considered when selecting models for HF research initiatives that are or may be significantly affected by these elements. Previous studies have examined cardiac remodeling in response to air pollution [133], circadian rhythms [134,135], and dietary interventions [136,137,138].

### 5.4. Gender and Age

Up-to-date, gender-related disparities in both health and disease are often overlooked in preclinical cardiac models. Gender and age are critical when modeling HF, as these factors influence pathophysiological mechanisms differently across the lifespan, illustrated by epidemiological differences demonstrated in variate HF subsets. HFpEF tends to dominate in women and progresses with age, whereas HFrEF is more prevalent in men [2]. *In vitro*, cell lines may be generated from either female or male patients, and both have been studied previously using monolayers of iPSC-derived CMs [139,140,141]. However, accurate interpretation and extrapolation of these results to explore gender-related disparities is hampered by the often-immature status of these cells [142,143] as well as the lack of relevant hormonal cues and challenges relating to X chromosome inactivation in female iPSC-CM derived constructs [144]. Overcoming these limitations may entail the development of more sophisticated cardiac tissue models, as well as the fine-tuning of media formulations [140] and genetic manipulation. In addition, the application of premature CMs *in vitro* and the use of mostly young animals *in vivo* greatly underestimates the contribution of age-dependent cardiac changes. Regarding HF, these changes may significantly affect cardiac reserve, quality of muscle contractility and relaxation due to matrix remodeling, the presence of comorbidities as well as the response to specific drug candidates. Recognizing the unique aspects of HF in different populations can improve *in vitro* model accuracy and accelerate the development of more effective and patient-specific therapies.

## 6. Challenges and Future Perspectives

The current challenges in cardiac tissue engineering that hinder clinical translation can be distilled into four core areas, covering all aspects of model design. Firstly, attaining *in vivo*-like mimicry of matrix biocompatibility and architecture, including blood, nerve, and lymphatic structures, remains ambitious due to the intricate composition of cardiac ECM. Current scaffolds often lack essential ECM proteins, deviate from native tissue function, and are static rather than dynamic like cardiac ECM. Vascularization and nerve integration are limited, as these systems require complex signaling and coordination that is hard to replicate, while the developmental course of lymphatic structures remains incompletely understood. Secondly, reaching full maturity in iPSC-derived multicellular cardiac constructs poses a significant challenge due to the complex developmental timeline of CMs, the need for precise environmental cues, and the difficulty in synchronizing the growth of multiple cell types with distinct requirements. Thirdly, fully replicating cardiac dynamic cues, including cyclic strain, electrical stimulation, and biochemical gradients appears difficult, as it requires synchronized, continuous stimuli across multiple dimensions. Specialized bioreactors are needed to provide controlled strain and electrical pacing while maintaining precise gradients of growth factors, hormones, and oxygen levels, which static culture systems lack. Integrating these factors into a single platform with long-term stability is technologically complex. Finally, developing multi-organ 3D engineered models presents additional challenges, primarily in harmonizing the distinct microenvironments, culture media, and dynamic stimuli that each organ system requires, which is difficult to recreate *in vivo*.

To tackle these challenges, research is focused on advancing biofabrication and 3D bioprinting techniques that integrate synthetic and biological materials into bioinks, enabling the creation of more accurate ECM mimics that incorporate vessel structures [145,146,147,148]. Additionally, dECM scaffolds are being increasingly explored to better replicate native cardiac environments with a specific focus on cardiac regeneration [149,150]. Coculture systems are being investigated to integrate multiple cell types, fostering enhanced cell-to-cell communication and further promoting cell maturation, while simultaneously driving the development of multi-organ-on-a-chip platforms [151,152]. Moreover, significant progress is being made in advancing bioreactor systems that provide synchronized mechanical and electrical stimulation to also support tissue maturation, alongside the development of microfluidic chip systems that offer precise control over dynamic conditions tailored to different tissue types [151,153,154].

Lastly, future *in vitro* HF research will increasingly focus on gender differences, as males and females show distinct patterns in disease progression and drug responses due to hormonal, genetic, and structural variations [140]. Researchers are turning to gender-specific iPSC models to study how hormones like estrogen and testosterone affect cardiac tissue [140]. Additionally, age-specific models will address how aging, including hormonal shifts in post-menopausal women, influences cardiac function and disease progression [155]. Integrating hormonal cycles and senescence will enhance understanding of how gender and age impact HF, leading to more personalized and effective treatments. Finally, advancements in 3D engineered cardiac models are moving toward more personalized approaches in general, using patient-specific iPSCs to capture unique genetic and molecular profiles. This personalization aims to replicate individual variability in disease mechanisms and drug responses, paving the way for tailored therapies that align more closely with each patient’s specific cardiac profile. While 3D engineered cardiac *in vitro* models provide unprecedented insights into cardiac physiology and disease mechanisms, inherent limitations may hinder their full translation to clinical science. Unlike whole-organ systems, 3D models can only approximate (patho)physiological conditions and multi-organ complexity, as well as immune interactions, and long-term adaptive responses that play critical roles in HF progression and treatment outcomes. Despite their human-derived cellular composition, these models currently offer predictive power that, while significant, does not yet fully surpass the established gold standard of animal models for clinical translation. However, given the limitations of animal models in accurately mirroring human biology, 3D engineered models serve as an essential complementary tool, enhancing predictive accuracy when used alongside other preclinical models in a sequential approach [14]. Moreover, 3D engineered cardiac models are increasingly suited for high-throughput applications, particularly with advancements in scalable microfluidic platforms [14]. These platforms excel in automated screening, managing multiple experimental conditions simultaneously, and providing a powerful solution for testing pharmacological effects and genetic interventions. As 3D cardiac engineered platforms become more sophisticated and standardized, there is hope that these integrated models will one day serve as a robust alternative to animal models for specific.

## 7. Conclusions

Accurate *in vitro* modeling using cardiac 3D engineered systems is essential to significantly advance HF research. These platforms range from simple single-cell cultures to sophisticated 3D constructs, organoids, and heart-on-a-chip systems, each offering unique insights and benefits. Key features impact the full scope of model design, ranging from extracellular matrix composition, architecture, mechanical properties, and electrical conductivity to the selection of appropriate cell types and ratios, as well as achieving cellular maturity. Incorporating dynamic modulation such as cyclic strain, fluid shear stress, and biochemical stimuli further enhances *in vivo* model mimicry. This review also underscores the relevance of inter-organ interactions, gender and age-related differences, and external environmental factors that could significantly improve predictive power in order to facilitate translation. 

Comprehensively, these models should ultimately enable therapeutic breakthroughs in HF research by answering critical fundamental questions while facilitating preclinical validation and improving the chances of successful clinical translation. Continued refinement and integration of new technologies will be key to overcoming HF challenges and advancing patient care.

## Figures and Tables

**Table 1:** Engineered 3D *In Vitro* Models for Modeling HF.

**Spheroids**	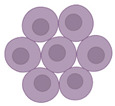
**Organoids**	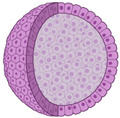
**Hydrogel models**	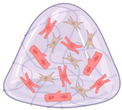
**Decellularized models**	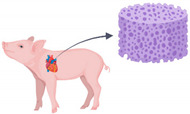
**Bioprinted tissues**	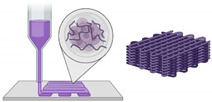
**Heart-on-a-chip models**	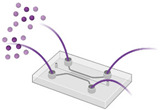

Created in BioRender.
